# Real-time risk ranking of emerging epidemics based on optimized moving average prediction limit—taking the COVID-19 pandemic as an example

**DOI:** 10.1186/s12889-023-15835-0

**Published:** 2023-06-01

**Authors:** Zhongliang Wang, Bin Liu, Jie Luan, Shanshan Lu, Zhijie Zhang, Jianbo Ba

**Affiliations:** 1 Naval Medical Center, Naval Medical University, Shanghai, China; 2 Department of Mathematics and Physics, Faculty of Military Medical Services, Naval Medical University, Shanghai, 200433 China; 3 Department of Epidemiology and Health Statistics, School of Public Health, Fudan University, Shanghai, China

**Keywords:** Op-MAPL, Risk ranking, Math model, COVID-19, SARS-CoV-2

## Abstract

**Background:**

Mathematical models to forecast the risk trend of the COVID-19 pandemic timely are of great significance to control the pandemic, but the requirement of manual operation and many parameters hinders their efficiency and value for application. This study aimed to establish a convenient and prompt one for monitoring emerging infectious diseases online and achieving risk assessment in real time.

**Methods:**

The Optimized Moving Average Prediction Limit (Op-MAPL) algorithm model analysed real-time COVID-19 data online and was validated using the data of the Delta variant in India and the Omicron in the United States. Then, the model was utilized to determine the infection risk level of the Omicron in Shanghai and Beijing.

**Results:**

The Op-MAPL model can predict the epidemic peak accurately. The daily risk ranking was stable and predictive, with an average accuracy of 87.85% within next 7 days. Early warning signals were issued for Shanghai and Beijing on February 28 and April 23, 2022, respectively. The two cities were rated as medium–high risk or above from March 27 to April 20 and from April 24 to May 5, indicating that the pandemic had entered a period of rapid increase. After April 21 and May 26, the risk level was downgraded to medium and became stable by the algorithm, indicating that the pandemic had been controlled well and mitigated gradually.

**Conclusions:**

The Op-MAPL relies on nothing but an indicator to assess the risk level of the COVID-19 pandemic with different data sources and granularities. This forward-looking method realizes real-time monitoring and early warning effectively to provide a valuable reference to prevent and control infectious diseases.

## Background

The disease surveillance systems based on open-source data from the Internet has attracted much more attention in public health, especially since the outbreak of the coronavirus disease 19 (COVID-19) pandemic caused by severe acute respiratory syndrome coronavirus 2 (SARS-CoV-2). The usage of Internet data makes it possible to achieve near real-time monitoring of infectious diseases and then the data is transformed to early-warning signals by models [1]. There are three common types of models, including the compartment model, the statistical model, and the machine learning model. The main modeling form of the compartment model is differential equations. The dynamics of the epidemic may change the behavior of its spread, which need to restructure and calibrate the model from the very beginning, and increases the complexity of model application [2]. The machine learning model, widely used in many fields such as healthcare and environment [3, 4], is considered as the most accurate in these three kinds of models, but require sufficient data to train and cannot clarify results based on causality [5]. It is difficult to fulfill syndromic surveillance timely and comprehensively in some regions, especially for developing countries, because of the limited sanitary condition [6]. Therefore, it is necessary to establish a statistical model as the core algorithm for the epidemic predication with certain accuracy and easy operation.

Statistical process control (SPC) is a mature and easy method. The Shewhart control chart, originally developed in an industrial process control setting, is an important tool of SPC and also used to detect disease outbreaks in prospective disease surveillance, with a disease outbreak coinciding with an out-of-control process. Based on Shewhart control chart, Page proposed the cumulative sum (CUSUM) control chart [7], which is able to detect small changes in the process mean that control charts more quickly. A typical application case based on long-term baseline data is salmonella outbreaks detected by the Centers for Disease Control (CDC) using the CUSUM method in 1995 [8]. The CUSUM based on short-term baseline data can be used under the lack of historical data, such as C1–MILD (C1), C2–MEDIUM (C2), and C3–ULTRA (C3) that are components of the Early Aberration Reporting System [9] and have detected outbreaks of West Nile fever and influenza [10]. Moreover, Karami et al. evaluated the performance of CUSUM algorithm in timely detection of meningitis outbreak with limited baseline data based on semi synthesis approach [11]. The CUSUM control chart was extended to Poisson count data [12] and used in many prospective surveillance applications [13], including surveillance systems such as Bio-Sense and the Electronic Surveillance System for the Early Notification of Community-based Epidemics [14].

The CUSUM can provide the information about the start time, length and severity of an outbreak. However, SARS-CoV-2 with various mutations resulted in seven pandemic peaks worldwide in the last two years and disturbed social formal operation, it is necessary of risk ranking during an outbreak to provide helpful reference for balance containment and international interactions. In view of this, He et al. proposed the moving average prediction limit (MAPL) methods which can assist in judging the epidemic trend of emerging infectious diseases and predicting the risk levels in a timely manner [15]. Epidemic districts or travelers may implement a differentiated precision control or prevention strategies respectively according to target location of epidemic risk. The MAPL can be regarded as an extension of CUSUM based on short-term baseline data. Specifically, the number of daily new cases was divided into five levels by using the four “prediction limits” of $\overline{x }\pm s$ and $\overline{x }\pm 2s$, and the corresponding risk score was given. Here, $\overline{x }$ and $s$ are mean and standard deviations based on the data from the past $T$ days. Calculate the moving average (MA) of the risk scores with respect to $T$, and then determine the risk level of the day.

Early-warning threshold is one of the important parameters of CUSUM, and its selection directly affects the detection power of the method [16]. Due to the natural trade-off between power and type I error rate, if the threshold is set too low, the false alarm rate will be increased; and if it is set too high, the outbreak point will be missed. The similar problem exists in the application of MAPL. If the prediction limits are too wide, the rapid rise of the epidemic cannot be captured in advance; and if the prediction limits are set too narrow, a slight fluctuation will lead to a high-level warning signal which loses the reference value of decision-making. In view of the above problems, this study first proposed and verified that the normalization of the number of newly diagnosed cases conformed to standard logistic distribution to improve the setting of prediction limits in MAPL method based on probability. Secondly, this study calculated MA for the number of normalization after truncation, not the risk score, which improves the utilization rate of the data and reduces the influence of outliers on epidemic trend judgment. Finally, in view of the characteristics of small data volume and large fluctuation before the outbreak, and changes in the transmission dynamics at the later stage of the epidemic, the optimized moving average prediction limit (Op-MAPL) method was proposed by combining the improved MAPL method with C1 (negative 1-sided CUSUM calculation) and C3 methods in order to mitigate the distortion of the evaluation with single index and realize full early-warning. The Op-MAPL can achieve the prediction of the peak day, certain predictability of risk ranking and high accuracy of prediction.

## Methods

### Data sources and processing

Data on the COVID-19 pandemic comes from the Github website (https://github.com/CSSEGISandData/COVID-19/tree/master/csse_covid_19_data/csse_covid_19_daily_reports), and its granularity is categorized by nation and province (or state, county) in two dimensions. Based on the monitoring and early-warning platform of COVID-19 established in Outbreaking Now System (OBN, http://27.115.41.130:8888/obn/) and the daily real-time diagnosed cases released from the Github website, the Op-MAPL model would run once on the daily update of the data source.

### Formulation of the Op-MAPL model

The Op-MAPL model is optimized based on the main point of the MAPL model and constructed as follows:

#### Trend value of the daily growth rate

In this paper, $T$ is the time interval, ${x}_{j}$ is the number of newly diagnosed cases of day $j$, and ${\overline{x} }_{j}$ denotes the sample mean of newly diagnosed cases in the $T$ observation days before day $j$:

1.1 $${\overline{x} }_{j}=\frac{1}{T} \sum_{i=j-T}^{j-1}{x}_{i}$$

${s}_{j}$ denotes the sample standard deviation of newly diagnosed cases in the $T$ observation days before day $j$:

1.2 $${s}_{j}=\sqrt{\frac{1}{T-1}\sum_{i=j-T}^{j-1}{\left({x}_{i}-{\overline{x} }_{j}\right)}^{2}}$$

${z}_{j}$ is defined as follows:

1.3 $${z}_{j}=\frac{{x}_{j}-{\overline{x} }_{j}}{{s}_{j}}$$

${z}_{j}$ is the normalization of newly diagnosed cases of the $T$ observation days before day $j$ and reflects the relative growth rate of newly diagnosed cases on day $j$.

Assume that ${z}_{j}$ conforms to the standard logistic distribution, and its probability density function and cumulative distribution function are as follows:

1.4 $$f\left(x\right)=\frac{{\mathrm{e}}^{-x}}{{\left(1+{\mathrm{e}}^{-x}\right)}^{2}}, F\left(x\right)=\frac{1}{1+{\mathrm{e}}^{-x}}$$

**Fig. 1:**
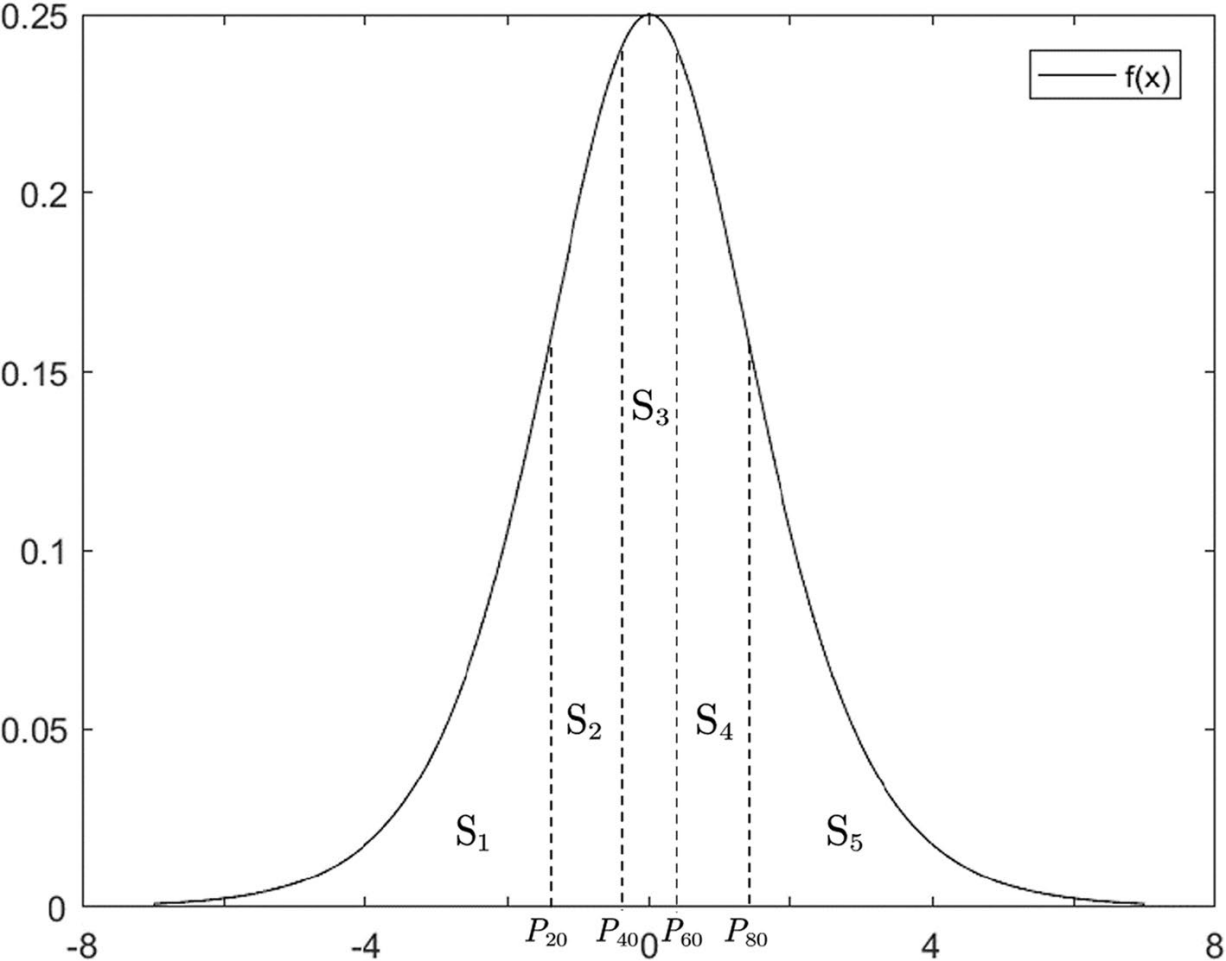
Standard logistic distribution probability density function curves and percentile sites of the area quintiles under the curve

${z}_{j}$ is classified based on probability as follows: divide the area (100%) under the curve of the probability density function $f\left(x\right)$ into five equal parts (Fig. 1). Let ${S}_{k}=20\%\left(k=1, 2,\cdots , 5\right)$, ${P}_{20}$, ${P}_{40}$, ${P}_{60}$ and ${P}_{80}$ are thresholds of 4 boundaries. ${P}_{i}$ means the $i$ th percentile. Solve the following equation:

1.5 $$F\left({P}_{k\times 20}\right)= \sum_{i=1}^{k}{S}_{i}$$

Obtain ${P}_{20}=-1.3863$, ${P}_{40}=-0.4055$, ${P}_{60}=0.4055$, ${P}_{80}=1.3863$. Take ${P}_{80}$ as an example. As $\left({z}_{j}>{P}_{80}\right)=1-F\left({x}_{4}\right)=0.2$, ${z}_{j}$ is in the top 20% of all possible values when ${z}_{j}>{P}_{80}$. Therefore, “${z}_{j}>{P}_{80}$” means that the number of newly diagnosed cases of day $j$ is in the “rapid growth” state. The detailed partition of ${z}_{j}$ is described in Table 1.

**Table 1:** Relative growth classification on day $j$

${\boldsymbol z}_{\mathbf j}$ values	Relative growth on day $\boldsymbol j$
${z}_{j}>{P}_{80}$	Rapid growth
${P}_{60}<{z}_{j}\le {P}_{80}$	Growth
${P}_{40}<{z}_{j}\le {P}_{60}$	Stability
${P}_{20}<{z}_{j}\le {P}_{40}$	Reduction
${z}_{j}\le {P}_{20}$	Rapid reduction

The ${z}_{j}$ value is susceptible to emergencies or various random factors and cannot accurately reflect the development trend of epidemics Therefore, the moving average of ${z}_{j}$ over the time interval is introduced:

1.6 $${\mathrm{MA}}_{j}=\frac{1}{T} \sum_{i=j-T+1}^{j}\mathrm{min}\left\{4, \mathrm{max}\left\{-4, {z}_{i}\right\}\right\}$$

${\mathrm{MA}}_{j}$ is the growth trend value on day $j$. To further decrease the interference of outliers, ${z}_{i}$ in (1.6) was truncated by $\pm 4$ for

1.7 $$P\left(-4<{z}_{j}\le 4\right)=F\left(4\right)-F\left(-4\right)=\frac{1}{1+{\mathrm{e}}^{-4}}-\frac{1}{1+{\mathrm{e}}^{4}}=0.964028.$$

This means $96.4\%$ chance of ${z}_{j}$ value in $\left(-4, 4]\right.$.

#### Indices for outbreak and significant risk-reduction

Because ${\mathrm{MA}}_{j}$ is the average of ${z}_{j}$, the classification described in Table 1 can also be used for ${\mathrm{MA}}_{j}$. However, there are three problems in practice: (1) it is only suitable for trend judgement and timely risk prediction after the epidemic has developed to a certain level [15] and (2) ${\mathrm{MA}}_{j}$ represents the relative growth rate. So only according to ${\mathrm{MA}}_{j}$, the epidemic risk ranking may be low, but newly diagnosed cases may be still high after the rapid decline over time. (3) The lag of evaluation.

In response to the above problems, we combine the C1 and C3 methods with ${\mathrm{MA}}_{j}$ to make comprehensive judgements.

For the first problem, we introduced a high sensitivity index ${\mathrm{C}3}_{j}$ in the early stage of the epidemic to decide whether the epidemic was in the outbreak state. The definition is as follows:

1.8 $${\mathrm{C}3}_{j}=\sum_{i=j-2}^{j}\mathrm{max}\left\{0, {\widetilde{z}}_{i}-K\right\}$$

$K$ represents the minimum deviation of the observed value from the expected value, and ${\widetilde{z}}_{i}=\frac{{x}_{i}-{\overline{x} }_{i-2}}{{s}_{i-2}}$ is the normalization of newly diagnosed cases on day $i$ with respect to the newly diagnosed cases in the $T$ observation days before day $j-2$. Compared with ${z}_{i}$, there is a 2-day buffer period between the baseline data and the current data in the definition of ${\widetilde{z}}_{i}$, thereby reducing the impact of the last two days [17, 18]. Referring to CDC Salmonella Outbreak Detection Algorithm [19], $K=1$ was set. The reference threshold of ${\mathrm{C}3}_{j}$ is 2 [20], that is, an outbreak is considered when ${\mathrm{C}3}_{j}\ge 2$.

For the second problem, we introduced a low-sensitivity index ${\mathrm{C}1}_{j}$ for detecting negative changes in the late stage of the epidemic from a "prudent" perspective, defined as follows:

1.9 $${\mathrm{C}1}_{j}=\left|\mathrm{min}\left\{0,{z}_{j}+K\right\}\right|$$

The definition of $K$ here is the same as above. The reference threshold for ${\mathrm{C}1}_{j}$ is 3 (referring to (1.7) and note $K=1$), which implies that it is considered that there is a “significant” reduction of newly diagnosed cases when ${\mathrm{C}1}_{j}\ge 3$, and that the epidemic has converted into a “medium–low risk” state.

For the third problem, we set the time interval $T=7$ considering the weekend effect on the data [10, 21, 22].

**Fig. 2:**
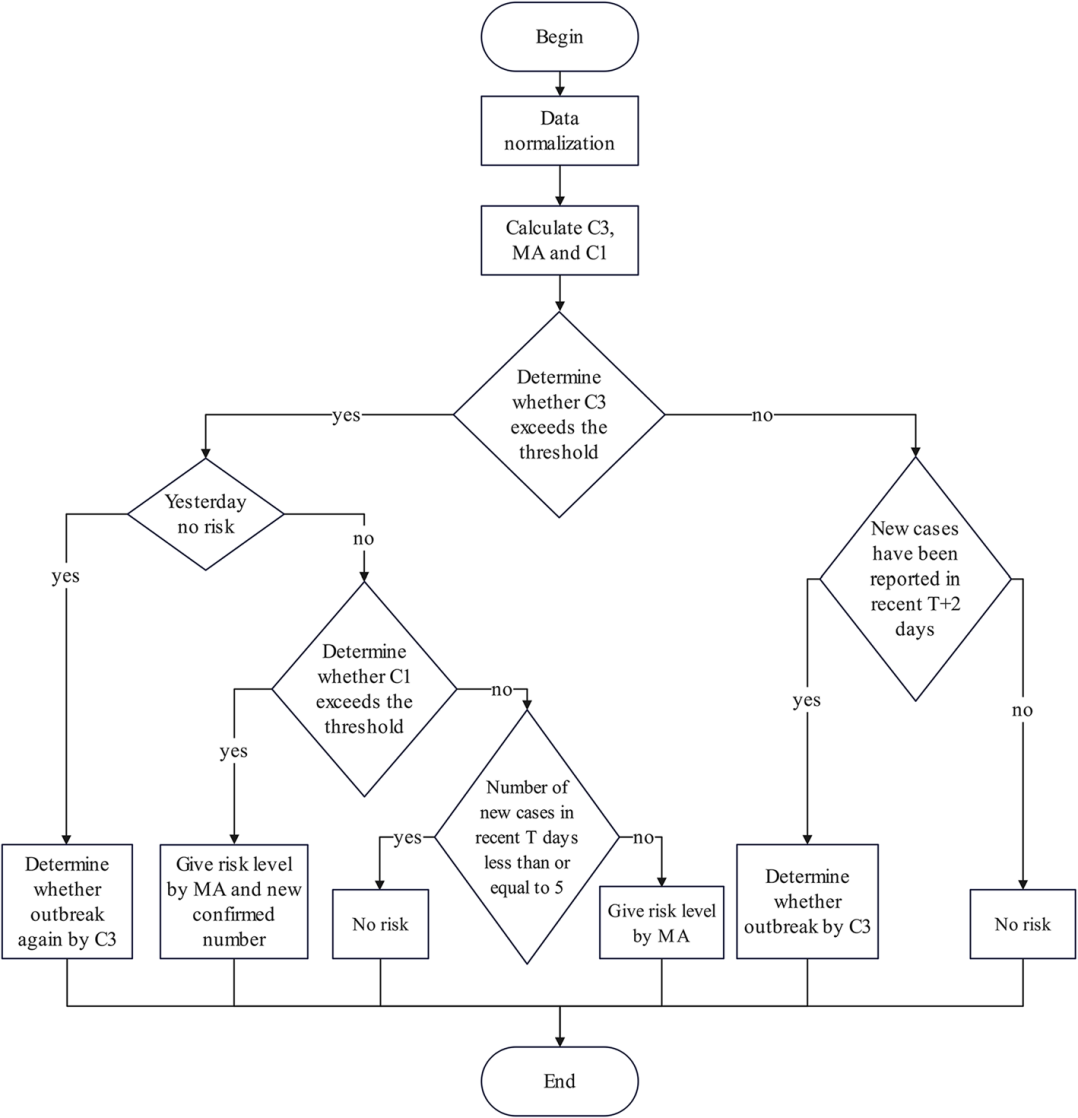
The flow chart of risk ranking process

Combining the above two indices and ${\mathrm{MA}}_{j}$, the real-time risk level description of the epidemic is given as shown in Table 2 and risk ranking process is showed in Fig. 2.

**Table 2:** The index range of risk classification of the epidemic

Index	Application stage	Prerequisites	Index range	Risk level description	Risk score
${\mathrm{C}3}_{j}$	Early	(1) Exist new cases in past 9 days (2) Day $j-1$ is no risk	${\mathrm{C}3}_{j}\ge 2$	Low-medium (outbreak)	2
${\mathrm{MA}}_{j}$	Whole	C3 signal	${P}_{40}<{\mathrm{MA}}_{j}\le {P}_{60}$	Medium	3
			${P}_{60}<{\mathrm{MA}}_{j}\le {P}_{80}$	Medium–high	4
			${\mathrm{MA}}_{j}>{P}_{80}$	High	5
		C1 signal	${P}_{20}<{\mathrm{MA}}_{j}\le {P}_{40}$ or ${\mathrm{MA}}_{j}\le {P}_{40}$, ${x}_{j}>100$ (1) ${\mathrm{MA}}_{j}\le {P}_{20}$ (2) $x_j\ \le\ 100$	Medium–low	2
				Low	1
${\mathrm{C}1}_{j}$	Late	(1) Risk score on day $j-1$ is 3 (2) ${\mathrm{MA}}_{j}\le {P}_{40}$	${\mathrm{C}1}_{j}\ge 3$	Low-medium (epidemic subsided)	2
${x}_{j}$	Whole	C3 signal	${x}_{j-\left(T-1\right), \cdots ,}$ ${x}_{j}\le 5$	No risk (shutdown C3 signal)	0

## Results

### Verification of the epidemic risk ranking based on the Op-MAPL model

#### Risk ranking for the pandemic peak of the Delta variant in India

The Delta variant of SARS-CoV-2 was first discovered in India in October 2020 [23]. India was found to be at the peak of the Delta round pandemic by observing past data. Therefore, February 15, 2021, when COVID-19 cases were at a relatively low growth rate, was selected as the starting point for observation. As the C3 method needs to accumulate data, the monitoring has been carrying out since February 6.

**Fig. 3:**
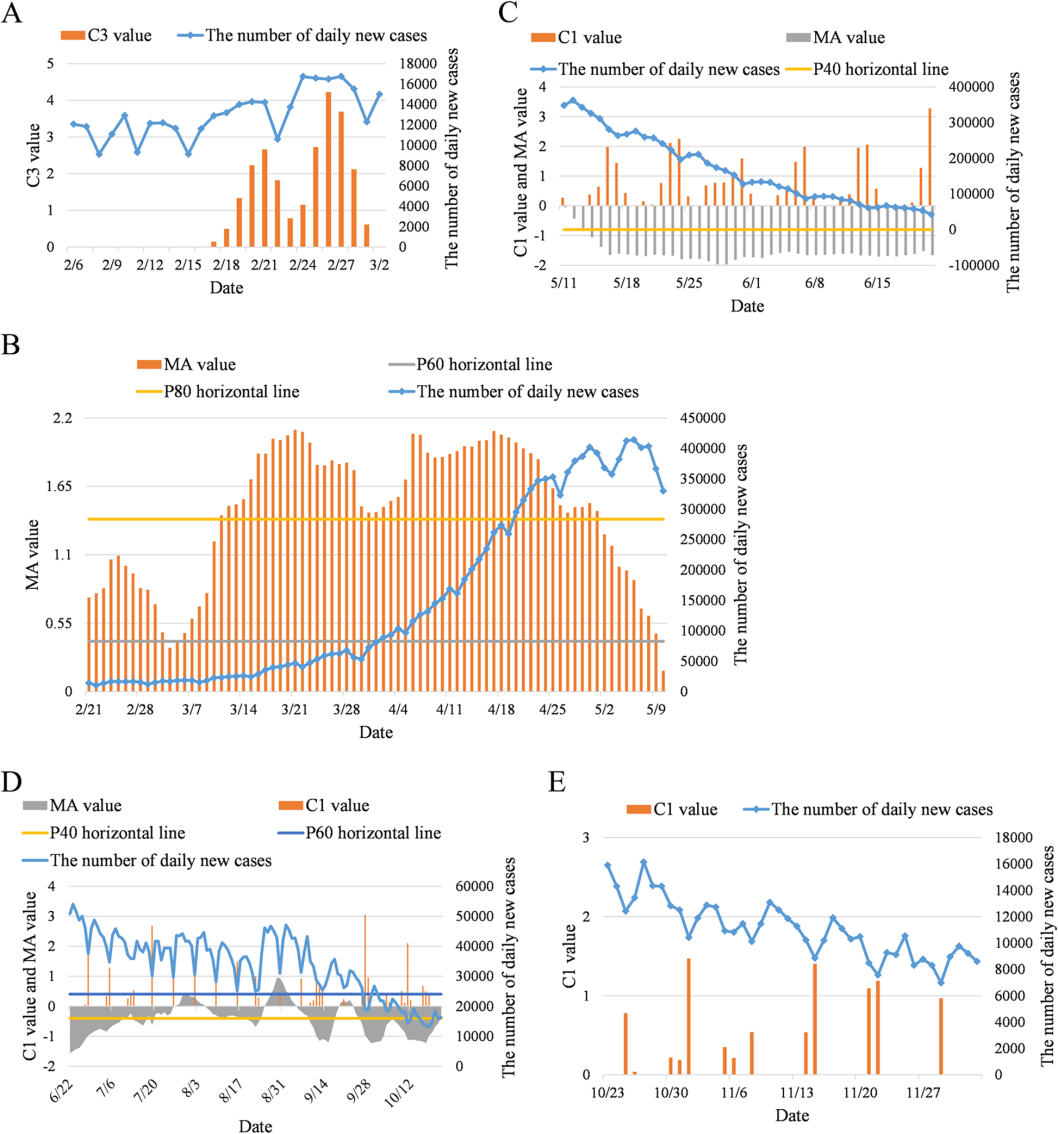
Risk ranking for the pandemic peak of the Delta strain in India. **A** C3 values from February 17 to March 2, 2021; **B** MA values of daily growth trend from February 21 to May 10, 2022; **C** C1 values and MA values of daily growth trend from May 11 to June 21, 2021; **D** C1 values and MA values of daily growth trend from June 22 to October 22, 2021; **E** C1 values from October 23 to December 3, 2021

As shown in Fig. 3A, the C3 value of February 17 was calculated after data accumulation for 9 days. The C3 value exceeded the threshold of 2 for the first time on February 20, which led to the release of an outbreak signal. Its risk level was assessed as medium–low (score 2) (Table 2).

Then, the risk levels were evaluated by the growth trend value ${\mathrm{MA}}_{j}$ from February 21 (Fig. 3B). The MA values exceeded the of ${P}_{60}$ on February 21, which would be assessed as “medium–high risk” (risk score 4) (Table 2). The MA values were between the ${P}_{60}$ and ${P}_{80}$ from February 21 to March 10 except for March 4 (Fig. 3B), which was still assessed as medium–high risk. The MA values exceeded the ${P}_{80}$ on March 11 (high risk, score 5). Meanwhile, the number of newly diagnosed cases increased remarkably compared with that of the previous days from March 15 (Fig. 3B), and the algorithm issued a “high-risk” warning 4 days in advance. The MA values fell below the horizontal threshold of ${P}_{80}$ on May 2, and the risk was lowered from high to medium–high. The growth of newly diagnosed cases slowed down, which meant that the pandemic was under control and indicated that the peak of the pandemic might be coming. The MA values decreased rapidly and fell below the ${P}_{80}$ on May 10 (medium risk) due to the slowdown of the new cases growth rate and high volatility.

The risk trend must be judged by the C1 values and the MA values after being rated as medium risk (Table 2). The MA values decreased below ${P}_{40}$ on May 15 (Fig. 3C), indicating that the new cases growth rate began to decline. However, newly diagnosed cases still increased at a high level which meant the risk level was still high. After that, the MA values remained below ${P}_{40}$ and continued downwards. The C1 value went below the threshold of 3 on June 21, and the risk was reduced to medium–low (Table 2). At this time, the number of newly diagnosed cases dropped by 1 order of magnitude compared to that of May 15.

The reduction rate of newly diagnosed cases has slowed since June 22 (Fig. 3D). The MA value exceeded the ${P}_{40}$ on July 10, and the risk rose to medium. Newly diagnosed cases fluctuated after that. On September 27, the C1 value once again went below the threshold of 3, and the risk returned to medium–low. Newly diagnosed cases began to go down again. On October 22, the MA value returned above ${P}_{40}$, and the algorithm issued a “medium risk” warning. Since then, as shown, although the increase in daily new cases still fluctuated downwards, the reduction rate slowed down significantly compared to that before October 22 (Fig. 3E). Because the C1 value did not exceed the threshold of 3 until December 2, the risk level has been kept at medium since October 22. A new round of outbreak occurred in India due to the Omicron strain emerging on December 2 [24].

#### Risk ranking for the pandemic peak of the Omicron variant in US

Omicron, a SARS-CoV-2 variant, was first detected in South Africa on November 9, 2021 [25]. The Omicron strain is more infectious and difficult to detect, and spreads faster than the Delta strain. In this section, the Omicron outbreak in the United States is analysed by the Op-MAPL method.

The first reported COVID-19 case concerning the Omicron strain appeared in the United States on December 1, 2021 [26], which was set as the starting point of surveillance.

**Fig. 4:**
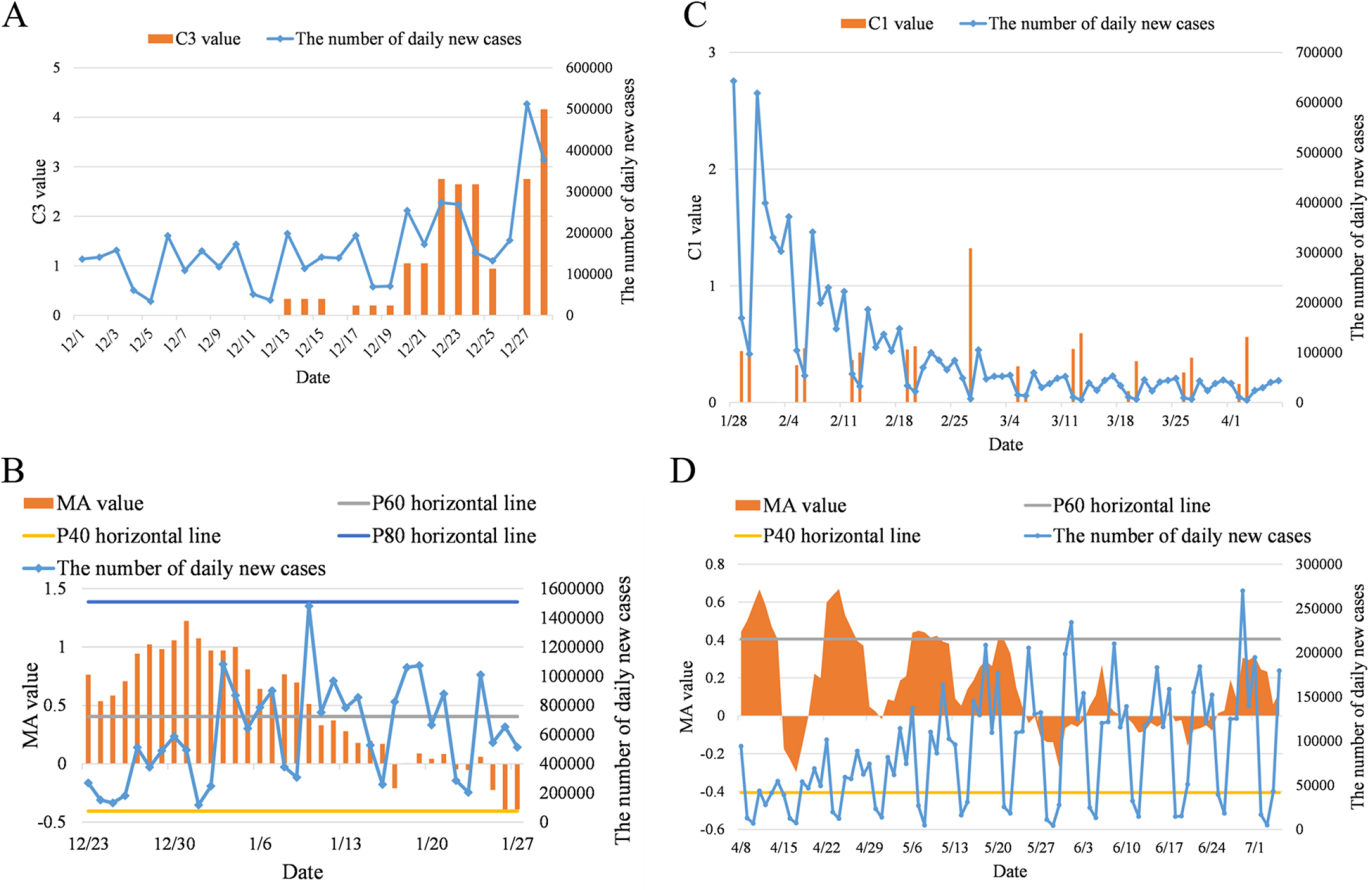
Assessment of the infection risk level of the pandemic peak of the Omicron variant in US. **A** C3 values from December 13 to December 28, 2021; **B** MA values of daily growth trend from December 23, 2021 to January 27, 2022; **C** C1 values from Jan 28 to April 7, 2022; **D** MA values of daily growth trend from April 8 to July 5, 2022

The C3 value exceeded the threshold of 2 on December 22, 2021 (Fig. 4A), indicating an outbreak of the pandemic, and the risk was rated as medium–low. Then, the MA value went over the ${P}_{60}$ on December 23 (Fig. 4B), entering the medium–high risk level and fluctuating until January 10, 2022. And the number of newly diagnosed cases increased and eventually reached a local maximum on January 10. The MA value fell below ${P}_{60}$ on January 11, which led to the risk being rated as medium. As of January 27, MA values remained between ${P}_{40}$ and ${P}_{60}$ with unchanged risk level. Meanwhile, the number of newly diagnosed cases fluctuated up and down with 600,000 as the midline.

Daily new cases have dropped significantly since January 28. However, the C1 value stayed under 3 until April 7 (Fig. 4C), so the risk remained at the medium level. The MA values from April 8 to 13, from April 22 to 26, and from May 6 to 10 were all greater than ${P}_{60}$ (Fig. 4D), and the algorithm issued a “medium–high risk” warning during these periods. The number of newly diagnosed cases during these periods was still relatively low with the observation of the pandemic later. Therefore, the 3 of medium–high risk assessment were considered as early warnings. The MA values were between ${P}_{40}$ and ${P}_{60}$ until July 5 except for these 3 periods, the risks during which were rated as medium.

#### Verification of epidemic risk ranking

The risk assessment of the epidemic in India and US is verified from the following five aspects.

##### Testing the assumption of ${z}_{j}$

**Fig. 5:**
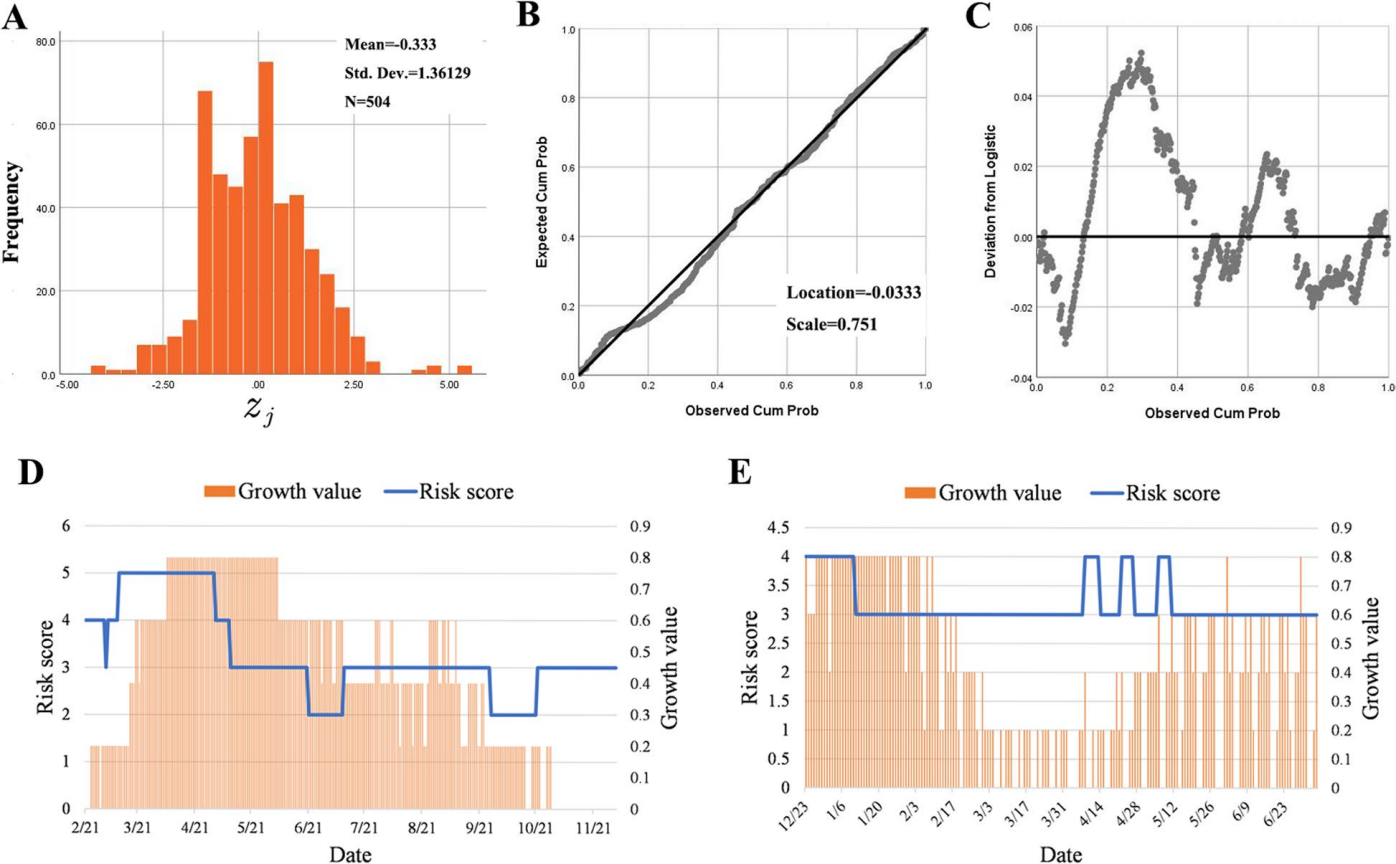
Distribution test of ${z}_{j}$ and predictability of risk ranking. **A** Histogram of ${z}_{j}$; **B** Logistic P-P Plot of ${z}_{j}$; **C** Detrended logsitic P-P Plot of ${z}_{j}$; The comparison between the risk score and the growth value in India (**D**) and US (**E**)

Since ${z}_{j}$ is a normalized result and not be affected by regional difference in theory, all the ${z}_{j}$ values of data in India and US can be merged and displayed in a histogram (Fig. 5A), which approximately conforms to standard logistic distribution compared. P-P plots were drawn for further verification: the actual distribution is basically close to the theoretical distribution (Fig. 5B) and the difference can be negligible according to the residual ranges from -0.03 to 0.05 (Fig. 5C). ${z}_{j}$ is usually regarded as conforming to the standard normal distribution in previous studies [9, 15, 17, 18, 20, 21]. Although the normal and logistic distributions are similar for a large sample, the later takes advantages in this study: the calculation of ${z}_{j}$ only involves a small sample ($T=7$) and the risk assessment is more accurate with standard logistic distribution; the length among 20th, 40th, 60th and 80th percentile of the standard logistic distribution is wider to make results more stable.

##### Peak prediction

Judging the epidemic peak timely is important to adjust the control and prevention measures, which can also be achieved by the Op-MAPL model. Because the daily risk ranking by the Op-MAPL reflect the epidemic growth rate recently, the decline of risk ranking with an increasing number of newly diagnosed cases indicates the potential arrival of the epidemic peak. The first day was considered as the predicted peak day when the risk score changed from 4 to 3. The predicted and actual peak days in India and US were showed in Table 3.

**Table 3:** Comparison of predicted peak and actual peak days in different countries

Country	Predicted peak days	Actual peak days
India (2021.02.21 -2021.12.03)	2021.5.10	2021.5.6
USA (2021.12.23–2022.07.05)	2022.1.11, 2022.4.14, 2022.4.27, 2022.5.11	2022.1.10

The time difference between the predicted and actual peak day in India is only 4 days. There are 4 predicted peak days in US because of local maximum predicted by this method when the epidemic fluctuated frequently. However, there is the largest increase of the number of newly diagnosed cases on January 11 2022 among the 4 predicted peak days of US, which is much close to the actual peak day.

##### Predictability of risk ranking

The document of newly diagnosed cases number is simplified in order to verify the early-warning function of the Op-MAPL. The number of newly diagnosed cases in India and the US in the observation period are ranked respectively to determine the 20th, 40th, 60th and 80th percentile. Growth value is defined according to Table 4.

**Table 4:** The relationship between the number of new cases and the growth value

Interval of newly diagnosed cases number	Growth value
$\ge {P}_{80}$	0.8
$\ge {P}_{60}$, $<{P}_{80}$	0.6
$\ge {P}_{40}$, $<{P}_{60}$	0.4
$\ge {P}_{20}$, $<{P}_{40}$	0.2
$<{P}_{20}$	0

Growth value can reflect relative increasing speed of newly diagoned cases number in the observation period. For example, the growth value 0.8 of day j indicates that the number of newly diagnosed cases of day $j$ is in the top 20% in the observation period. The relationship between daily risk score and growth value is showed in Fig. 5D and 5E. It is clear that the risk scores issued by Op-MAPL are always ahead of the development of the epidemic.

##### Accuracy of prediction

The risk scores truly not only reflect the average growth rate of the epidemic in the past T days, but also can predict the subsequent risk scores due to the application of the moving average method. Here is risk score comparison of one day and the days later one by one (Table 5). The risk score accuracy of predicting the 6th and 7th days in US was below 80% due to the large fluctuation of the data and others are high. This means that risk raking by the Op-MAPL model is stable and indeed reflects the short-term trend of the epidemic.

**Table 5:** The accuracy of risk score prediction of post days

Days later	1	2	3	4	5	6	7
India	96.84%	94.37%	91.87%	89.36%	86.83%	84.29%	82.08%
USA	96.39%	92.75%	89.06%	85.34%	81.58%	79.89%	79.26%

### Application of the Op-MAPL model to the current Omicron variant in China

#### Shanghai

**Fig. 6:**
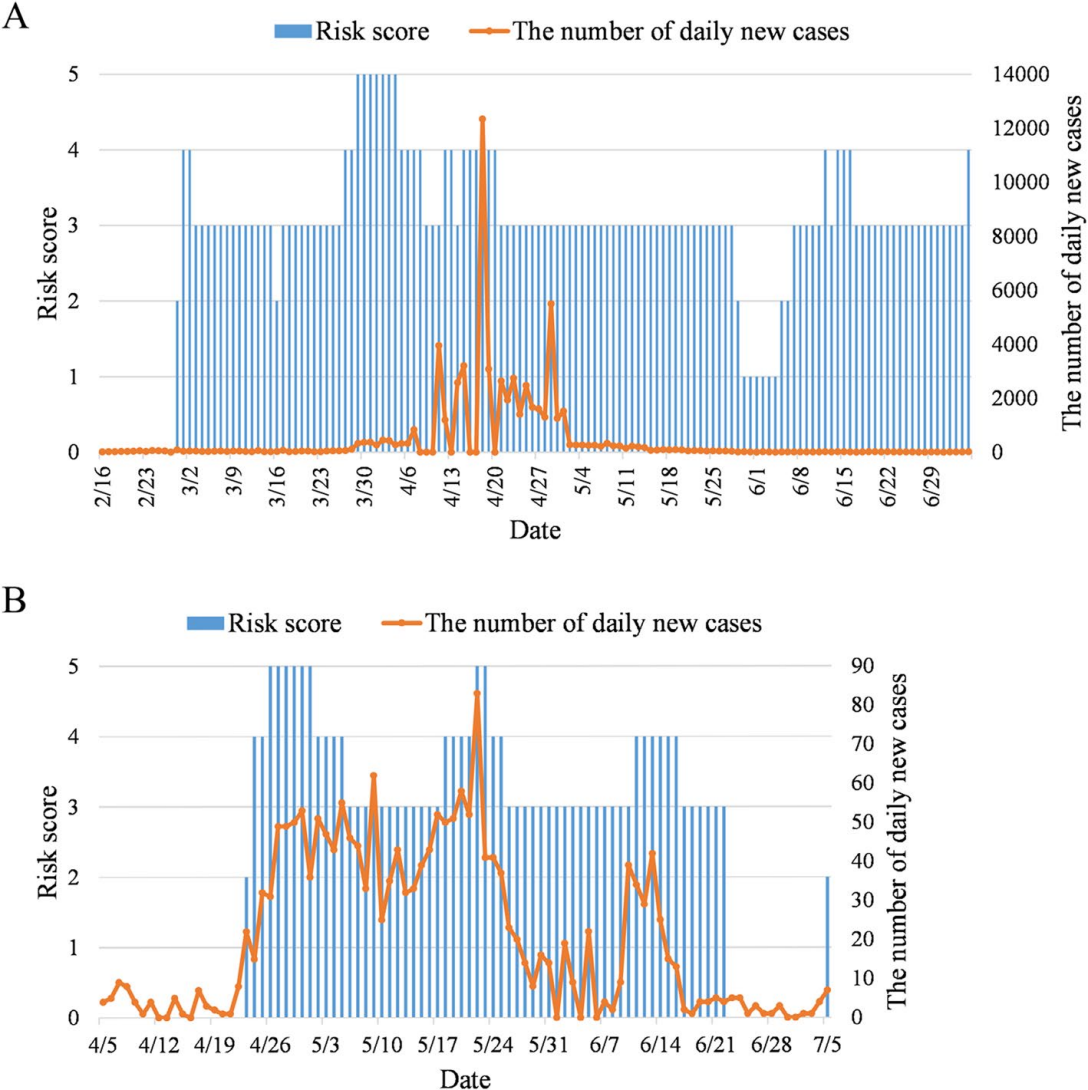
Risk scores of the pandemic in China. Shanghai from Feb 28 to July 5, 2022 (**A**) and Beijing from April 23 to July 5, 2022 (**B**)

An indigenous COVID-19 case was diagnosed in Putuo District, Shanghai, on March 1, 2022, and the first case was confirmed to be infected by the Omicron variant after gene sequencing analysis on March 7 [27]. Taking data accumulation into account, February 16 was regarded as the starting point for monitoring. The Shanghai pandemic risk assessments before July 5 are shown (Fig. 6A).

The Op-MAPL showed that the outbreak occurred on February 28 and was rated as medium–low risk. On March 1 and 2, the risk level was medium–high, and immediately fell back to medium on March 3. The risk level was then kept at medium until March 26. A 4-day strict lockdown was imposed on Shanghai Pudong New District on March 28. The Op-MAPL issued a “medium–high risk” warning on March 27 and upgraded it to a “high risk” warning on March 29. Puxi District ended the first round of 4-day lockdowns on April 5, and the algorithm showed that the risk level has downregulated as medium–high. Hereafter, Shanghai was under strict lockdown for nearly 2 months. The algorithm always prompted medium–high risk until April 20 except for a few short periods. On April 21, the algorithm assessed the risk level as medium, indicating that the pandemic has been brought under control. From Shanghai's official data, it can be concluded that the pandemic reached peak before April 21. Although there were repetitions of the pandemic, newly diagnosed cases decreased in a downwards trend. Meanwhile, the algorithm continued to prompt medium risk level until May 28. It showed that the risk has downregulated as medium–low on May 29. The number of newly diagnosed cases decreased to 100, and the MA was less than ${P}_{20}$ from May 30 to June 4, which led to the risk level being rated as low (score 1) and indicated the end of the pandemic in Shanghai. In fact, Shanghai has already fully resumed work and production since June 1.

The MA value went above ${P}_{20}$ on June 5, and the rating of the risk level was adjusted to medium–low. The algorithm rated the risk level to medium on June 7 as the decreasing rate slowed down with new cases. The potential infection risk increased on June 9 after three new social cases were confirmed. On June 12, the algorithm prompted medium–high risk through calculation. After the city's urgent screening and quarantine, the pandemic was effectively controlled, and mass infection was avoided. On June 17, the algorithm rated the risk level back to medium. However, a clustered outbreak on July 3 resulted in the algorithm issuing a “medium–high risk” warning again on July 5.

#### Beijing

Another indigenous COVID-19 case in Beijing was confirmed on April 22, and the pandemic had spread for a week already [28]. Considering the need for data accumulation, the starting point of testing was moved forward to April 5 and the risk evaluation before July 5 was shown in Fig. 6B.

The Op-MAPL released the outbreak signal on April 23 and rated it as low-medium risk. Due to the rapid increase of newly diagnosed cases, a “high-risk” warning was issued on May 26. Then the pandemic trend moved downwards under control, so the risk level fell into the medium on May 6 and stayed until May 17. Subsequently, the pandemic recurred, and the algorithm responded with a “high-risk” warning quickly on May 22 at the peak of this round. The pandemic spread was blocked again, so the number of newly diagnosed cases returned to a low level and the risk level returned to medium on May 26. Then the newly diagnosed cases fluctuated at a low level, so the medium risk level kept until June 10.

A cluster outbreak occurred in many places on June 9 and aggravated the spread risk. The algorithm rated medium–high risk level on June 11. The growth rate of newly diagnosed cases abated on June 16 due to the effective anti-pandemic measures and a “medium risk” warning was issued again on June 17. The number of newly diagnosed cases in 7 consecutive days was less than 5 by June 23, so the risk was downwards to score 0 and turned off the C3 signal, marking the end of the pandemic. However, the C3 value exceeded the threshold of 2 on July 5, and a “medium–low risk” warning was issued again.

## Discussion

Risk classification is necessary to assist decision-making to balance containment and international interactions for worldwide infectious diseases. In this study, the Op-MAPL method is proposed to achieved real-time risk ranking for the COVID-19 pandemic of different SARS-CoV-2 variants in different regions, and verified. Results showed that the predicted epidemic peaks are very closed to actual peaks, the daily risk ranking is stable and predictive, and the average accuracy of classification prediction within 7 days was 87.85%. In addition, there are five aspects further discussed as follow.

### The rationality of assuming that ${{\varvec{z}}}_{{\varvec{j}}}$ follows the standard logistic distribution

The SI model, which is one of the classical compartment models, includes three assumptions: (1) the total number of people remains unchanged; (2) there are only susceptible and infected people in the population; and (3) someone can be infected if he (she) has effective contact with infected people. The solution of the SI model is as follows:

2.1 $$i\left(t\right)=\frac{1}{1+\left(\frac{1}{{i}_{0}}-1\right){\mathrm{e}}^{-\uplambda t}},$$

$i$ represents the proportion of infected people in total, *t* represents time, *i*_0_ is the proportion of infected people at the initial moment (*t* = 0), and $\uplambda$ represents the number of effective contacts of each infected person per unit time. Equation (2.1) shows that the number of newly diagnosed cases conforms to the logistic distribution under assumptions (1)-(3).

This paper sets the time interval *T* = 7, that is, the period is one week. First, the population change of the country or region within a week is negligible, so model assumption (1) is satisfied. Second, it is reasonable to neglect the case of recovery and reinfection due to the short time interval (*T* = 7). Latent infected persons with infectivity are not counted in the cumulative number of infections, which make a lag in statistics. However, this case exists continuously and leaves no effect on the simulation of cumulative number of infections. Therefore, it can be considered that there are only two types of people—the susceptible and the infected. Finally, human factors, such as control, prevention and vaccine injections, have hysteresis and stability for their effects. Therefore, the "infection environment" faced by the public within a week is relatively unchanged, so the standard of “effective contact” is uniform.

Based on the discussion of the above two aspects, the daily numbers of newly diagnosed cases in every 7 days can be considered to approximately conform to the logistic distribution with the same parameters. Therefore, from the definition of *z*_*j*_, *z*_*j*_ follows the standard logistic distribution. This is the theoretical basis to improve the prediction limits in this study, and the introduction of *z*_*j*_ reduces the loss of information in the process of epidemic risk assessment.

### False alarms and amendments

The surveillance of emerging infectious diseases is a continuous process. The introduction of the index C3_*j*_ allows us to assign a value to show the infection risk, which greatly avoids the unreal “high risk” assessment caused by the fluctuation of new cases in the early stage of the epidemic and alleviates the need for MAPL to track the development of the epidemic to a certain level. However, false alarms may also be issued by the C3*j* value [20]. This paper continues to use the traditional setting of the threshold in C3*j* [20], but because *z*_*j*_ follows the standard logistic distribution, the probability of *z*_*j*_ falling within -3 to 3 (notice K = 1) is only 90.5%. Therefore, a threshold of 2 may be more sensitive in actual detection, but it is necessary to do so based on the principle of "prudence". In this study, the outbreak signal of Beijing on July 5 sent by the Op-MAPL is a false alarm in practice. However, a certain amount of historical data has been accumulated, the growth trend value MA_*j*_ can stably reflect the trend of the epidemic, which can correct false alarms quickly with the help of the C1_*j*_ value.

### Rationality of the introduction of index C1_*j*_

When the epidemic has reached peak and the number of newly diagnosed cases drops rapidly, it is not appropriate to judge the epidemic trend merely based on MA_*j*_ because the risk assessment may be low but the newly diagnosed cases are still high. Therefore, this study introduced C1_*j*_ in the late stage of the epidemic for risk trend judgement.

The mechanism of the C1, C2 and C3 methods is to determine whether “abnormal” growth exists in the early stage of the epidemic, which is the basis for judging the outbreak. This study assumes that the number of newly diagnosed cases conforms to the logistic distribution, so there will theoretically be an abnormal “drop point” of the number of newly diagnosed cases symmetrical to the “outbreak point” of the epidemic with the help of the symmetry of the distribution (Fig. 1). Therefore, this study innovatively uses the C1 method, which is less sensitive among the three methods, to define a negative change detection index C1_*j*_ to capture the “drop point” and to alleviate the risk ranking declining too fast.

However, the C1 signal does not appear in every round of the epidemic due to various factors. Therefore, from a “prudent” point of view, this study allows a longer-term “medium risk” assessment for risk classification. Moreover, the risk level will be revised based on the number of newly diagnosed cases. For example, Beijing's pandemic risk on June 23 was directly reduced from a medium level to no risk because it met the conditions that there are no more than 5 daily new cases in 7 consecutive days.

### The selection of the time interval

Time interval is always selected by the periodicity of infectious diseases when using the traditional moving average method [29]. Judgements can only be made based on the incubation period for emerging infectious diseases due to the uncertainty of the infection cycle [15]. The trend evaluation results may be seriously delayed if time interval is selected too long, or they may be easily disturbed by fluctuations and cannot reflect the trend well if it is selected too short. After comprehensive consideration, this study selects the time interval *T* = 7. In addition to satisfying the assumption of *z*_*j*_, it also considers the basic time unit of human activity. From this article, we found that this time interval can not only reduce the interference but also respond to the epidemic trend sensitively regardless of the epidemic scale.

### Limitations of Op-MAPL

Because some factors, such as individual immunity, are not considered and the proportion of newly diagnosed cases during the quarantine in the later stage of the epidemic is relatively high, there may be autocorrelation between the data. Therefore, judgements based solely on the classification standards in Table 2 may cause deviations in the later stage of the epidemic, and other methods must be combined. Comparing with the MAPL, the Op-MAPL introduces new indices to assist judgement. They are integrated into a unified framework but increases the complexity of risk classification. The classification standard should be further simplified through theoretical derivation. In addition, the criteria in the classification below medium risk are more rigid than those above medium risk. For example, although *x*_*j*_ ≤ 100 in the low risk level is based on the consideration of community transmission, this standard can be more flexible for different granularity data and should be combined with local basic characteristics, such as population density and topographical condition.

## Data Availability

The datasets generated and/or analysed during the current study are available in the the Github website, https://github.com/CSSEGISandData/COVID-19/tree/master/csse_covid_19_data/csse_covid_19_daily_reports. All data is obtained from the corresponding author on reasonable request.

## Abbreviations

Op-MAPL: Optimized Moving Average Prediction Limit
COVID-19: Coronavirus disease 19
SARS-CoV-2: Severe acute respiratory syndrome coronavirus 2
SPC: Statistical process control
CUSUM: Cumulative sum
OBN: Outbreaking Now System
MAPL: Moving average prediction limit
MA: Moving average
CDC: Centers for Disease Control
